# Lay health supporters aided by mobile text messaging to improve adherence, symptoms, and functioning among people with schizophrenia in a resource-poor community in rural China (LEAN): A randomized controlled trial

**DOI:** 10.1371/journal.pmed.1002785

**Published:** 2019-04-23

**Authors:** Dong (Roman) Xu, Shuiyuan Xiao, Hua He, Eric D. Caine, Stephen Gloyd, Jane Simoni, James P. Hughes, Juan Nie, Meijuan Lin, Wenjun He, Yeqing Yuan, Wenjie Gong

**Affiliations:** 1 Sun Yat-sen Global Health Institute, School of Public Health and Institute of National Governance, Sun Yat-sen University, Guangzhou, Guangdong, China; 2 Xiangya School of Public Health, Central South University, Changsha, Hunan, China; 3 Department of Epidemiology, School of Public Health and Tropical Medicine, Tulane University, New Orleans, Louisiana, United States of America; 4 Department of Psychiatry, University of Rochester Medical Center, Rochester, New York, United States of America; 5 Department of Global Health, University of Washington, Seattle, Washington, United States of America; 6 Department of Psychology, University of Washington, Seattle, Washington, United States of America; 7 Department of Biostatistics, University of Washington, Seattle, Washington, United States of America; 8 Silver School of Social Work, New York University, New York, New York, United States of America; Harvard Medical School, UNITED STATES

## Abstract

**Background:**

Schizophrenia is a leading cause of disability, and a shift from facility- to community-based care has been proposed to meet the resource challenges of mental healthcare in low- and middle-income countries. We hypothesized that the addition of mobile texting would improve schizophrenia care in a resource-poor community setting compared with a community-based free-medicine program alone.

**Methods and findings:**

In this 2-arm randomized controlled trial, 278 community-dwelling villagers (patient participants) were randomly selected from people with schizophrenia from 9 townships of Hunan, China, and were randomized 1:1 into 2 groups. The program participants were recruited between May 1, 2015, and August 31, 2015, and the intervention and follow-up took place between December 15, 2015, and July 1, 2016. Baseline characteristics of the 2 groups were similar. The patients were on average 46 years of age, had 7 years of education, had a duration of schizophrenia of 18 years with minimal to mild symptoms and nearly one-fifth loss of functioning, and were mostly living with family (95%) and had low incomes. Both the intervention and the control groups received a nationwide community-based mental health program that provided free antipsychotic medications. The patient participants in the intervention group also received LEAN (Lay health supporters, E-platform, Award, and iNtegration), a program that featured recruitment of a lay health supporter and text messages for medication reminders, health education, monitoring of early signs of relapses, and facilitated linkage to primary healthcare. The primary outcome was medication adherence (proportion of dosages taken) assessed by 2 unannounced home-based pill counts 30 days apart at the 6-month endpoint. The secondary and other outcomes included patient symptoms, functioning, relapses, re-hospitalizations, death for any reason, wandering away without notifying anyone, violence against others, damaging goods, and suicide. Intent-to-treat analysis was used. Missing data were handled with multiple imputations. In total, 271 out of 278 patient participants were successfully followed up for outcome assessment. Medication adherence was 0.48 in the control group and 0.61 in the intervention group (adjusted mean difference [AMD] 0.12 [95% CI 0.03 to 0.22]; *p =* 0.013; effect size 0.38). Among secondary and other outcomes we noted substantial reduction in the risk of relapse (26 [21.7%] of 120 interventional participants versus 40 [34.2%] of 117 controls; relative risk 0.63 [95% CI 0.42 to 0.97]; number needed to treat [NNT] 8.0) and re-hospitalization (9 [7.3%] of 123 interventional participants versus 25 [20.5%] of 122 controls; relative risk 0.36 [95% CI 0.17 to 0.73]; NNT 7.6). The program showed no statistical difference in all other outcomes. During the course of the program, 2 participants in the intervention group and 1 in the control group died. The limitations of the study include its lack of a full economic analysis, lack of individual tailoring of the text messages, the relatively short 6-month follow-up, and the generalizability constraint of the Chinese context.

**Conclusions:**

The addition of texting to patients and their lay health supporters in a resource-poor community setting was more effective than a free-medicine program alone in improving medication adherence and reducing relapses and re-hospitalizations. Future studies may test the effectiveness of customization of the texting to individual patients.

**Trial registration:**

Chinese Clinical Trial Registry ChiCTR-ICR-15006053.

## Abstract 2

Wenjie Gong and colleagues show that mobile messaging can increase medication adherence and reduce relapses in schizophrenia patients in rural China.

## Author summary

**Why was this study done?:**

- Schizophrenia is a leading cause of disability, but in low- and middle-income countries (LMICs) the treatment gap remains high, and even when treatment is available, adherence to antipsychotics is low.
- Mobile text messaging has been shown to be useful to strengthen community- and family-based care in resource-poor settings due to its availability, reliability, and ease of use.
- However, there has been no clear evidence that texting improves treatment adherence, symptoms, and functioning in people with schizophrenia.

**What did the researchers do and find?:**

- We developed an intervention called LEAN, involving the recruitment of lay health supporters; an electronic platform with mobile texting for medication reminders, health education, and monitoring; the awarding of token gifts for positive behavioral improvement, and integration of the texting with the health system.
- In this 2-arm randomized controlled trial, the control group received a free antipsychotic medication program (the 686 Program) while the intervention group received the 686 Program plus LEAN.
- We found significant improvement in medication adherence (0.48 in the control group versus 0.61 in the intervention group) and a substantial reduction in the risks of relapse and re-hospitalization with the intervention.

**What do these findings mean?:**

- LEAN demonstrated a 27% improvement in adherence, which is larger than the 15%–18% range reported in other text message interventions.
- Two unique features may have contributed to the relative superiority of LEAN: active engagement of lay health supporters and the use of texting to connect the entire treatment team from patients to lay health supporters to village doctors to psychiatrists, all in support of the patient.
- Texting effectively addressed the low adherence at marginal cost, and LEAN’s experiences may be useful for other LMICs with resource constraints.
- However, an adaptation of LEAN should fully consider some implementation details including keeping the program simple and integrated into routine care, maintaining low cost, and choosing existing texting platforms if available.

## Introduction

Schizophrenia is a leading cause of disability, with a global prevalence of 0.4% [1] and contributing to 1.7% of total years lived with disability [2]. Schizophrenia also leads to a high economic burden [3] and the violation of human rights due to the stigmatization of the illness [4] and other causes. The WHO’s Mental Health Gap Action Programme (mhGAP) has identified schizophrenia as the top priority for global action, recommending treatment with antipsychotic medicines and psychosocial care [5]. However, in low- and middle-income countries (LMICs) the treatment gap remains high [6–8], and even when treatment is available, adherence to antipsychotics is low compared with adherence to drugs for other diseases because of patients’ lack of insight into their illness, their forgetfulness due to cognitive impairment, and side effects that are often associated with schizophrenia [9,10]. In LMICs, with limited mental health facilities and the healthcare workforce concentrated in large urban centers, the scarcity, inequity, and inefficiency of mental health resources present challenges [11,12]. As a result, there is a broad consensus for collaborative stepped care that emphasizes community- and family-based treatment, task sharing among human resources, and integrating mental health into existing primary healthcare [5,7,13].

In community- and family-based healthcare, mobile health (mHealth) has gained increasing traction [14–16]. With the proliferation of mHealth studies, there is a tendency to pursue “advanced” and often complicated mHealth solutions instead of simple but reliable methods that may work better among poorly educated populations in low-resource settings. Short message service (SMS) text messaging, or texting, as a simple and technologically reliable method, has been shown to be particularly useful in resource-poor settings due to its wide availability, reliability, ease of use, and relatively low cost [15,17]. Since the first publication of studies involving texting for health in 2002 [18,19], text messaging has been found to benefit diabetes self-management, weight loss, physical activity, smoking cessation, and medication adherence to antiretroviral therapy [17,20]. For people with serious mental disorders such as schizophrenia, texting has been used in 4 areas of application: reminders for medication and clinical appointments [21,22], information dissemination, supportive messaging, and self-monitoring procedures [15]. However, despite the recent proliferation of text messaging, there has been no clear evidence that technology-based prompts improve treatment adherence, symptoms, or functioning in people with schizophrenia [23,24]. Most studies to date have been small pilot studies that focused on feasibility rather than health outcomes [25,26], were primarily conducted in high-income countries [15,25], did not include informal caregivers (who often play important roles in schizophrenia management) [27], and often served as a stand-alone intervention not integrated with the health system [28]. Also, these studies paid insufficient attention to user evaluation and appreciation of the program [20] and to establishing a theoretical basis or working mechanism for the mobile intervention [29].

The LEAN intervention was intended to address some of the insufficiencies of the previous trials mentioned above and to have broad applicability for resource-poor settings in LMICs. This study was conducted as a pragmatic trial to test our primary hypothesis that lay health supporters (family members of the patients or community volunteers) aided by a simple texting system would increase patient adherence to antipsychotic medications, and improve symptoms and functioning in a community-based cohort of patients.

## Methods

### Study design and participants

We designed a 2-arm individually based randomized controlled trial. Details of the study design, methods, and analysis plan have previously been published as a study protocol [30]. The trial was prospectively registered in the Chinese Clinical Trial Registry (ChiCTR-ICR-15006053). Individual rather than cluster design was used as the likelihood of contamination or spillover effects of the intervention was considered minimal due to the private nature of our texting and tendency of patients and their families in rural China not to discuss mental conditions publicly. The study recruited and followed-up the patients between May 1, 2015, and July 1, 2016, in 9 rural townships (population 356,900) of Liuyang Municipality, Hunan Province, in central China.

We applied minimal inclusion and exclusion criteria [30]. The inclusion criteria were (1) being community-dwelling, (2) being an enrollee of the National Continuing Management and Intervention Program for Psychoses, known as the “686 Program,” (3) having a primary diagnosis of schizophrenia, (3) being on oral psychotropic medications, and (4) physically residing in 1 of 9 rural townships of Liuyang Municipality. People were excluded if they (1) were hospitalized due to schizophrenia at the time of recruitment (our intervention required sustained community residence), (2) had missed the most recent 3 consecutive past drug refills (in this case, they had de facto dropped out of the 686 Program), or (3) were physically incapable of using voice and text messaging (hearing and/or vision impairment prevented the use of our intervention). Trial participants were selected by simple random sampling from the 686 Program registry, which included almost all known villagers diagnosed with schizophrenia in Liuyang. The diagnosis of schizophrenia in the 686 Program used ICD-10 [31]. Trained interviewers obtained written informed consent from both the patient and the lay health supporters.

### Procedures

The development of the intervention, LEAN (Lay health supporters, E-platform, Award, and iNtegration), was guided by theory, empirical evidence, and our trial and error. In early 2015, challenged by low medication adherence among 686 Program enrollees, we piloted a program in rural Liuyang that tasked “village doctors” (paraprofessionals with rudimentary medical training) to directly deliver and monitor medication ingestion in patient homes [32]. However, the village doctors were already overburdened and had neither the time nor the incentive to take on more work. After rounds of consultation with policy-makers, clinicians, and patients and their families, we reached a consensus that only a low-cost, low-burden, easy-to-implement, and easy-to-use intervention was acceptable. Subsequently, following an adapted health belief model (HBM) [33], we selected individual components of LEAN from empirical literature on “task sharing,” medication adherence, and mHealth to improve patient adherence to medication. According to the HBM, people with schizophrenia may adhere to medications if they are properly “cued” to action (e.g., by prompts from the LEAN text messages and the lay health supporters to take medications) after weighing the net benefits of the medication against the perceived threat of schizophrenia (e.g., LEAN texted messages to improve patients’ understanding of the benefits of medication and the consequences of not controlling schizophrenia, and to enhance their handling of relapse and side effects) (details elsewhere [30]).

The acronym LEAN summarizes the 4 program components: Lay health supporters, E-platform, Award, and iNtegration. A lay health supporter was selected from the patient family or the community, who followed phone-texted instructions to perform simple tasks: supervising patient medication, monitoring side effects and relapse, and facilitating urgent care. We approached the people in the following order in identifying the lay health supporters: (1) the main family caregivers registered on the 686 Program registry, (2) family members accompanying the patients to collect medication refills, (3) family members nominated by the patients, and (4) community volunteers nominated by the mental health administrator with the agreement from the patients. The e-platform (an existing commercial telemarketing system) texted 2 daily messages to both the patients and their lay health supporters: a message at 9:00 AM with educational information on schizophrenia and a reminder at 7:00 PM to take medicine (see S2 Appendix for sample messages). All messages were phrased to be caring, polite, and personal as those characteristics were patient-preferred. To reduce user fatigue, the medication reminder was embedded in a message about local weather and news (e.g., “Good evening. Tomorrow: Sunny, 23 degrees. Go out and enjoy the Temple Fairs in ABC village. Please text 1 after taking medicine.”). We also sent occasional messages with a 14-item checklist for early signs of relapse [34] and medication side effects. The lay health supporter was expected to text back “1” if any item was checked, to which a project coordinator would follow up with a phone call. A group of master’s and doctoral students in public health and medicine were tasked to produce the messages, mainly adapting contents from evidence-based sources. A senior psychiatrist reviewed and approved messages for use. Every week our project coordinator prepared a texting report based on the messaging server data that included a list of families who texted back more frequently than the past month to confirm the taking of medication. The mental health administrators used the lists to award this improvement with a token gift such as a bar of soap and a congratulatory text message on a monthly basis. Finally, text messaging also served as a communication tool that integrated the efforts of lay health supporters into the existing health system—one example of this being an arrangement between village doctors, the project coordinator, and patients’ psychiatrists that if signs of relapse were detected, village doctors were texted to assess severity and the project coordinator then scheduled an appointment with the psychiatrist and texted appointment details to the patient’s family.

The patients in the intervention group and their lay health supporters received training in preparation for LEAN. The lay health supporters received a brief introduction of their responsibilities and roles at the time of recruitment, a demonstration on phone use (for those who had not had a phone and were given a free phone), and continued training through our text messages on how to provide patient care and seek professional help. The patients were evaluated for their ability to use 3 basic phone functions: turning the phone on/off, charging the phone, and reading/returning text messages. The patients in need of training received up to 3 sessions (20 minutes per session) of hands-on demonstration on how to use their phones (details elsewhere [35]).

After a 3-month pilot of LEAN, we made several design and implementation changes. Among the major changes, we decided to text medication reminders to both the lay health supporters and the patients rather than the patients alone, and to use an existing telemarketing platform rather than the one we developed on our own (the existing platform was less expensive, easier to implement, and technologically more sophisticated).

While the intervention group received the 686 Program plus LEAN, the control group received the 686 Program alone. The 686 Program is a national public program covering 5.4 million people with psychosis in China, three-fourths of whom are people with schizophrenia [36]. Despite some local variations, the basic structure of the 686 Program remains the same across the country. In Liuyang, a psychiatrist served as the full-time program director, supported by 2 other psychiatrists and several staff members working part-time (the psychiatrists were internists who converted their roles through on-the-job training). The psychiatrists together with several staff members traveled every 2 months to each township health center (THC) to provide patient consultations and free medication. The township mental health administrators, supervised by both the psychiatrists and the local THCs, coordinated the work of the village doctors to provide regular services, which included yearly physical exams, assessment of risk level, ≥4 home visits throughout the year, health education, and urgent care.

### Randomization and masking

The 686 Program registry included as high a proportion of villagers with schizophrenia as possible. We first selected 400 names from the registry with simple random sampling. Then we applied inclusion and exclusion criteria to this group and recruited those eligible after obtaining their informed consent. After the patients were recruited, a statistician not otherwise associated with the project allocated participants (1:1) by simple randomization to receive either the 686 Program plus LEAN (intervention group) or the 686 Program alone (control group). It was not possible to blind the program participants to allocation. However, outcome assessors were blind to group assignment and were physically separated from the program implementation team, which operated LEAN and monitored user experiences. The psychiatrists were also blind to the allocation status of participants. If unmasking occurred inadvertently during assessment, this was to be reported immediately, and a make-up assessment scheduled with separate assessors.

### Outcomes

Our published trial protocol provided details on measurements [30]. In brief, the primary outcome was a score of adherence to antipsychotic medications—the proportion of dosages taken over the past 30 days. The measurement was primarily based on unannounced home-based pill counts [37], and we followed the consensus guideline of including both objective and subjective measures when assessing adherence [38–40]. The pill counts were unannounced to minimize a potential “Hawthorne effect” [41]. To obtain 1 measurement of adherence, our evaluators made 2 home-based pill counts 30 days apart at the fifth month and the sixth month, and the difference between the 2 counts was considered the number of pills taken. We developed specific procedures to handle multiple bottles, discarded pills, and additionally acquired pills in between the 2 counts. The number of pills prescribed for that period was obtained from the 686 Program medication prescribing system. Adherence was calculated as (number of first count − number of second count + number of additional pills obtained − number of pills discarded) ÷ (number of pills prescribed). The counts were unannounced in the sense that although patients knew we would assess their adherence, they did not know on which home visit we would count pills, as village doctors scheduled those visits with the 686 Program routine visits whenever possible. To supplement pill counts, we also measured adherence through adherence rating scales (Brief Adherence Rating Scale [BARS] [42] and the Drug Attitude Inventory–10 [DAI-10] [43]) and medication refill records. We did not use electronic medicine caps to measure adherence as they provide a form of adherence intervention in themselves and do not improve the accuracy of measurement above that of unannounced pill counts. Due to resource constraints, we used refill records, DAI-10, and BARS instead of pill counts for the measurement of the baseline adherence. The rationale, validity, and details of our methods to measure adherence to antipsychotics in LEAN were published and are publicly available elsewhere [44]. Secondary outcomes were patient symptoms measured by the Clinical Global Impression (CGI) for schizophrenia (which includes 2 scales: CGI–Severity and CGI–Improvement) [45] and patient functioning measured by the WHO Disability Assessment Schedule 2.0 (WHODAS) [46]. The 686 Program psychiatrists administered CGI on patients, and the trained public health master’s and doctoral students assessed adherence and functioning. Outcomes were assessed at baseline and 6 months. The 686 Program registry provided additional data on patient attendance for clinical appointments, relapses (defined as an overall and marked increase in symptoms assessed by the health professionals through interviewing patients and family members according to the 686 Program protocol), re-hospitalization due to schizophrenia, and incidence of death for any reason, wandering, violence against others, damaging goods, and suicide. We also captured information on program cost and user experiences from various program operation channels and patient surveys. At baseline, we used the Glasgow Antipsychotic Side-effect Scale (GASS) to assess patient-reported side effects of antipsychotics, but we did not assess side effects as an outcome during follow-up due to a program administrative mishap. All data were double-entered into and managed by REDCap, a web-based secured data management tool [47].

### Statistical analysis

Using adherence data based on the clinician impression from the 686 Program management system, we determined that an increase of medication adherence from 0.72 to 0.85 (SD 0.33) would be a minimally important difference after consultation with 686 Program policy-makers. Following a standard procedure for a hypothesis of equal population means based on *t* test, we calculated that a total sample size of 258 participants (129 per group) would have 85% power to detect an increase of medication adherence from 0.72 to 0.85, assuming a 5% type I error and 10% attrition. All analyses including the subgroup analyses were conducted as pre-specified in the protocol [30]. Statistical package R was used.

We first performed a descriptive analysis of the data: sociodemographic information, key covariates, and outcomes at baseline were compared between the intervention and control groups to assess the randomization and participant characteristics. For the analysis of the program effect, intent-to-treat analysis was used for all participants. Missing outcomes were imputed based on demographic information and some other outcomes using the R package MICE [48], and 10 sets of data for each outcome were imputed (S1 Appendix). We used a semi-parametric generalized estimating equation (GEE) model to analyze program effect on adherence, symptoms, and functioning (i.e., medication adherence scores, WHODAS scores, and CGI–Severity scores, respectively) at the endpoint. We used GEE instead of analysis of covariance (ANCOVA) as initially proposed on our protocol due to a potential violation of the normal distribution assumption required for ANCOVA. Adherence analysis was adjusted for baseline adherence, WHODAS, and CGI scores; substance use; medication side effects; and family supervision, all of which are empirically suggested strong baseline predictors of adherence and pre-specified in our protocol. WHODAS and CGI analyses were adjusted for baseline WHODAS and CGI scores, respectively. We used the same GEE models for the analyses of 2 subgroups identified by medication refill adherence over the past year at baseline (people who collected all 6 refills were considered adherent, and those who missed any of the 6 refills were considered nonadherent) and functioning (cutoff at 0.22). To enable cross-study comparison, we calculated the program effect size as Cohen’s *d* [49].

### Ethical approval

The study protocol was approved by the institutional review boards of the University of Washington (49464 G) in Seattle, Washington, US, and Central South University (CTXY-150002-6) in Hunan, China. All patients and lay health supporters in this study gave their written informed consent before taking part in the study.

## Results

### Participant profile

**Fig 1:**
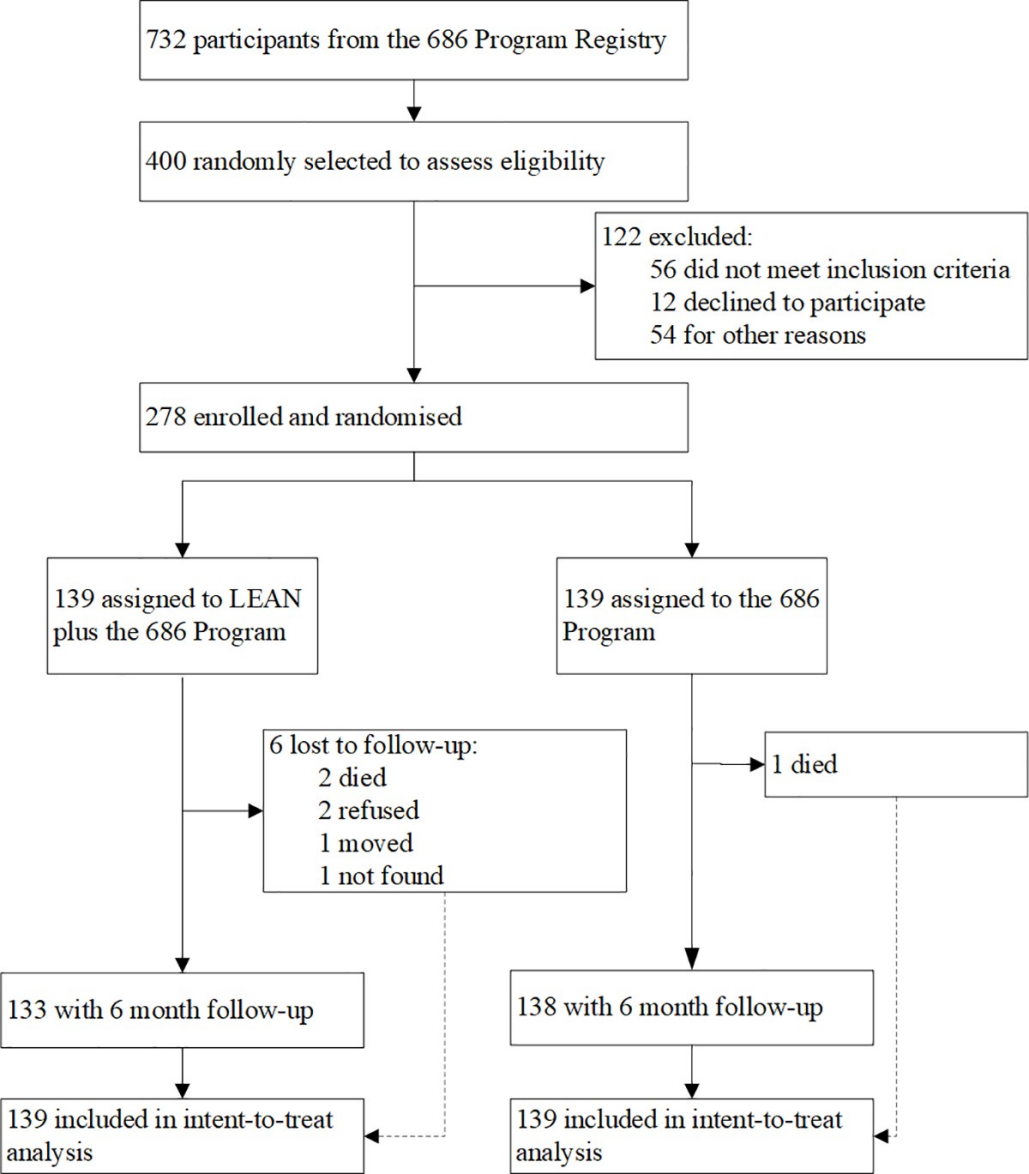
Trial profile.

Fig 1 shows the trial profile. Due to a higher rate of signing up to the program than expected, we recruited 278 patients out of the 400 candidates randomly selected from the 686 Program registry, slightly more than what we had planned. These 278 enrolled patients were randomized 1:1 into the intervention and control groups. Among the 400 candidates we approached to recruit, 12 people refused the enrollment, among whom most did not provide specific reasons for refusal; 56 (14%) did not satisfy our inclusion/exclusion criteria (e.g., some were not eligible as their primary diagnosis was epilepsy or another mental condition rather than schizophrenia); and 54 were not successfully contacted for consent due to various reasons (e.g., wrong contact information in the registry or not available/present at the time of our recruitment visits). The recruitment of the program participants occurred between May 1, 2015, and August 31, 2015, and the program was piloted from September 1 to November 30, 2015. The official intervention and follow-up took place between December 15, 2015, and July 1, 2016. Six participants (4%) from the intervention group and 1 (0.7%) from the control group were lost to follow-up at the 6-month assessment. No outcome interviews were unmasked throughout the trial. The sociodemographic and clinical profiles were comparable between the intervention and control groups at baseline (Table 1) (see S3 Appendix for free medications dispensed by the 686 Program). The patients were on average 46 years of age, had 7 years of education, had a duration of schizophrenia of 18 years with minimal to mild symptoms and nearly one-fifth loss of functioning, and mostly lived with family (95%) and had low incomes. Each patient in the intervention group was successfully assigned a lay health supporter: 80.6% of the lay health supporters were family members (mostly spouses and parents), and the remaining were community volunteers (Table 1).

**Table 1:** Baseline characteristics.

Characteristic	Count (%) or mean (SD)	
	Intervention (*n =* 139)	Control (*n =* 139)
**Patients**		
Female	77 (55.4%)	77 (55.4%)
Married	87 (62.6%)	90 (64.8%)
Employed	44 (31.7%)	48 (34.5%)
Living alone	7 (5.0%)	6 (4.3%)
Age (years)	46.5 (12.65)	45.5 (12.72)
Education (years)	7.4 (3.28)	7.1 (3.22)
Literate^a^	121 (87.1%)	126 (90.6%)
Patient income last month (RMB)^b^	66 (0–500)	95 (0–800)
Family annual income (RMB)^b^	20,000 (10,000–50,000)	20,000 (10,000–50,000)
Duration of schizophrenia (years)	17.5 (10.36)	18.4 (10.82)
**Caregivers/lay health supporters**^c^		
Female	67 (48.2%)	67 (48.2%)
Age (years)	45.4 (12.75)	44.5 (12.49)
Employed	76 (54.7%)	62 (44.6%)
Family member of the patient	112 (80.6%)	109 (78.4%)
Married	77 (55.4%)	76 (54.7%)
**Patients’ health profile**		
Medication adherence^d^		
Refill record^e^	0.75 (0.30)	0.71 (0.34)
Drug Attitude Inventory–10 (DAI-10)^f^	0.65 (0.20)	0.68 (0.20)
Brief Adherence Rating Scale (BARS)^g^	0.73 (0.18)	0.70 (0.20)
Clinical Global Impression (CGI)–Severity^h^	2.95 (1.66)	3.09 (1.70)
WHO Disability Assessment Schedule 2.0 (WHODAS)^i^	0.18 (0.19)	0.19 (0.18)
Glasgow Antipsychotic Side-effect Scale (GASS)^j^	9.47 (6.66)	8.59 (8.02)
Top 5 antipsychotics prescribed		
Clozapine	48/136 (35.3%)	45/133 (33.8%)
Risperidone	46/136 (33.8%)	43/133 (32.3%)
Quetiapine	26/136 (19.1%)	25/133 (18.8%)
Sulpiride	21/136 (15.4%)	25/133 (18.8%)
Perphenazine	12/136 (8.8%)	15/133 (11.3%)

^a^Literate: defined as no less than 3 years of primary school education.

^b^Indicated as median (IQR). RMB, renminbi.

^c^For the intervention group, these caregivers were recruited as “lay health supporters.”

^d^Per our research protocol, medication adherence measured by 2 unannounced home pill counts 30 days apart at endpoint was used for the analysis of program effect; however, pill counts were not performed at baseline. Instead, refill records and 2 rating scales were used at baseline.

^e^Adherence by refill record was calculated as a cumulative medication possession ratio (0%–100%) over 1 year, i.e., number of days medication obtained over 365 days divided by 365 days.

^f^DAI-10 adherence was originally from −10 to +10 (higher scoreequals more positive attitude toward medication), which was rescaled to be 0 to 1.

^g^BARS adherence is self-reported percentage of dosages taken over the past month.

^h^Higher scores of CGI–Severity indicate worse symptoms (possible range 1–7).

^i^WHODAS scores indicate the proportion of functioning lost.

^j^GASS scores indicate patient-reported side effects of antipsychotics: 0–21, no/mild side effects; 22–42, medium side effects; 43 and above, serious side effects.

### Process indicators

We collected a range of process indicators related to training, program implementation, user experiences, and content of family care. For training, we were able to assess 103 out of the 139 patients in the intervention group for their ability to use the phone: 72 (69.9%) patients were deemed in need of training, and 62 patients subsequently received the training. In total, 29 (46.8%) trainees were capable of using the 3 phone functions (turning the phone on/off, charging the phone, and reading/returning text messages) after the training (more details elsewhere [35]). Information on phone ownership and maintenance, the frequency of phone number changes, and users’ experiences and satisfaction are summarized in Table 2. In total, 58 (41.7%) patients and one-fifith of lay health supporters did not have a phone and received a free phone with US$15 prepaid data. In total, 62 out of 63 patients and 77 out of 77 lay health supporters expressed satisfaction with the program, although we cannot conclude that the participants were overall satisfied due to a large amount of missing data (Table 2). Following the same protocol, 8 master’s and doctoral students in public health produced a total of 237 educational text messages that covered self-care, medications, symptoms, relapse prevention, rehabilitation, and social resources; 2 messages on relapse signs and medication side effects; and about 150 unique messages of reminders (S2 Appendix). Overall, 27.0% of the families (lay health supporters and/or patients) responded to the medication reminders by texting back “1” every day; 47.0% responded at least once per week. Over the 6 months of follow-up, LEAN cost a total of RMB 53,500 (US$7,926) for the 139 patient participants and 139 lay health supporters in the intervention group, which included RMB 19,000 (US$2,815) for texting fees, RMB 7,600 (US$1,126) for the message development, RMB 4,800 (US$711) for the message management, RMB 10,000 (US$1,481) for the 77 phones provided to the patients and the lay health supporters, and RMB 9,000 (US$1,333) for the additional time cost for the health workers.

**Table 2:** User experiences in the intervention group.

Experience	Patients (*n =* 139)^a^	Lay health supporters (*n =* 139)^b^
**Phone status**		
Used a smartphone	33/114 (29.0%)	35/105 (33.3%)
Free phone given by LEAN	58/139 (41.7%)	19/139 (13.7%)
Changed phone numbers over past 2 months	13/105 (12.4%)	92/100 (92.0%)
Phones fully functioning at endpoint	77/99 (77.8%)	92/100 (92.0%)
**User evaluation at endpoint**		
Overall satisfied with the program	62/63 (98.4%)	77/77 (100.0%)
Willing to continue receiving messages	52/57 (91.2%)	80/85 (94.1%)
Messages very useful	61/103 (59.1%)	47/78 (60.3%)
Messages bothered you	4/63 (6.3%)	9/84 (10.7%)
Time of texting appropriate	57/62 (91.9%)	70/77 (90.9%)
Frequency of texting appropriate	53/61 (86. 9%)	66/79 (83.5%)
Length of messages appropriate	59/60 (98.3%)	71/77 (92.2%)
Most useful part of the messages		
Treatment and medication education	10/59 (17.0%)	18/73 (24.7%)
Family care in schizophrenia	5/86 (8.5%)	8/73 (11.0%)
Medication reminders	27/59 (45.8%)	39/73 (53.4%)
Local news	2/59 (3.4%)	1/71 (1.4%)
Weather forecast	15/59 (25.4%)	7/73 (9.6%)
**User capability assessed at endpoint**		
Able to navigate phone to read messages	52/73 (71.2%)	74/88 (84.1%)
Able to reply to messages	38/73 (52.1%)	55/86 (64.0%)
Did not understand messages	12/68 (17.7%)	44/90 (4.9%)
Some physical disability that prevents using a phone	12/65 (18.5%)	9/84 (10.7%)
**User experiences assessed at endpoint**		
Always received messages last month	44/71 (62.0%)	65/84 (77.4%)
Always or often read messages	39/71 (54.9%)	65/85 (76.5%)
Frequently replied to texted reminders	15/67 (22.4%)	27/85 (31.8%)
Were concerned about the cost of messages	7/64 (10.9%)	4/83 (4.8%)

^a^Patients: the patients in the intervention group of the program.

^b^Lay health supporters: the lay health supporters for the participants in the intervention group.

### Primary outcome: Adherence

**Fig 2:**
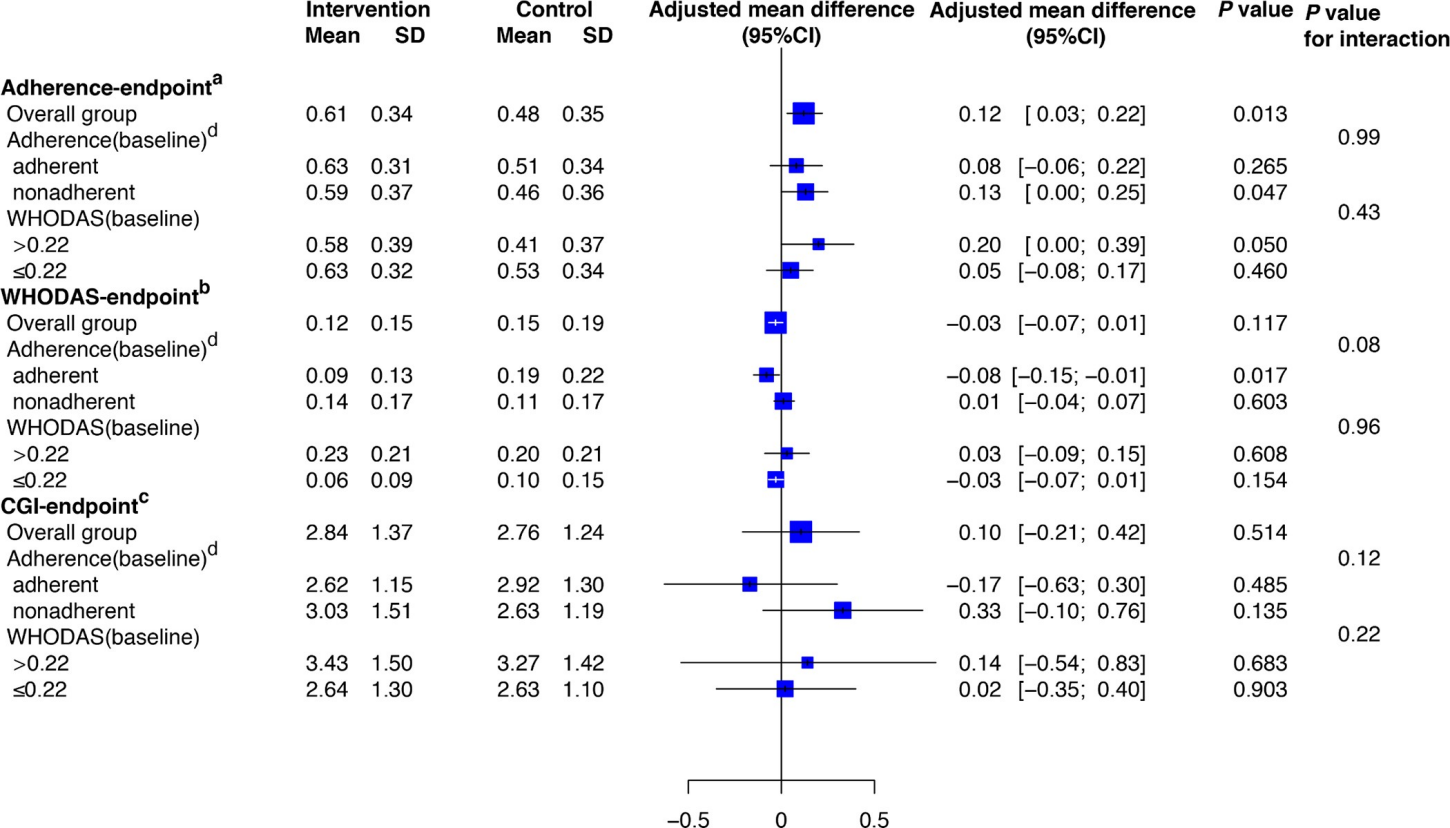
Subgroup analysis. ^a^Adherence endpoint is proportion of antipsychotic dosages taken over a month assessed by 2 unannounced home-based pill counts 30 days apart at 6 months (possible range 0–1). ^b^WHODAS endpoint is WHO Disability Assessment Schedule 2.0, indicating proportion of loss of functioning at 6 months (possible range 0–1). ^c^CGI endpoint is Clinical Global Impression–Severity assessed at 6 months (possible range 1–7). ^d^Adherence (baseline) is based on medication refill adherence over the past year at baseline: people who collected all 6 refills were considered adherent, and those who missed any of the 6 refills were considered nonadherent.

We note strong evidence of an intervention effect on adherence to antipsychotic medications. Medication adherence measured by the unannounced home-based pill counts was 27% greater in the intervention group (0.61) than in the control group (0.48) (adjusted mean difference [AMD] 0.12 [95% CI 0.03 to 0.22]; *p =* 0.013; effect size 0.38; Table 3; Fig 2). Our study was underpowered to detect treatment interaction with baseline adherence (*p* for interaction 0.99), although we found a similar intervention effect on adherence within the subset who were nonadherent at baseline (mean adherence at endpoint 0.59 in the intervention group versus 0.46 in the control group; AMD 0.13 [95% CI 0.00 to 0.25]; *p =* 0.047; effect size 0.36; Table 3; Fig 2), while the program effect attenuated for the baseline adherent group (AMD 0.08 [95% CI -0.06 to 0.22]; *p =* 0.265). In total, 59 out of 278 participants (21.22%) had missing outcomes (S1 Appendix). Meanwhile, the participants in LEAN attended a mean of 83% of scheduled clinical appointments, higher than the 76% in the control group (*p =* 0.066).

**Table 3:** Primary and secondary outcomes at 6 months.

Measure	Mean (SD) or *n/N* (%)		Mean difference (95% CI) or relative risk (95% CI)	*p*-Value
	Intervention (*n =* 139)	Control (*n =* 139)		
**Primary outcome**				
Pill-count adherence^a^	0.61 (0.34)	0.48 (0.35)	0.12 (0.03 to 0.22)^b^	0.013
Other adherence measurements				
DAI-10^c^	0.68 (0.20)	0.67 (0.22)	0.02 (−0.05 to 0.08)	0.67
BARS^d^	0.71 (0.21)	0.68 (0.23)	0.03 (−0.04 to 0.10)	0.37
Refill record^e^	0.83 (0.28)	0.76 (0.34)	0.04 (−0.01 to 0.10)	0.12
**Secondary outcomes**				
WHODAS^f^	0.12 (0.15)	0.15 (0.19)	−0.03 (−0.07 to 0.01)^b^	0.117
CGI–Severity^g^	2.84 (1.37)	2.76 (1.24)	0.11 (−0.21 to 0.42)^b^	0.514
Negative	2.94 (1.46)	2.98 (1.43)	0.02 (−0.29 to 0.32)	0.908
Positive	2.70 (1.62)	2.67 (1.55)	0.17 (−0.14 to 0.49)	0.277
Depression	2.31 (1.29)	2.11 (1.26)	0.75 (−0.15 to 0.30)	0.517
Cognition	2.86 (1.50)	2.85 (1.44)	0.07 (−0.22 to 0.36)	0.617
CGI–Improvement^h^	3.09 (1.15)	3.02 (1.08)	0.03 (−0.25 to 0.30)	0.848
**Other outcomes from the “686 Program”**^i^				
Relapse^j^	26/120 (21.7%)	40/117 (34.2%)	0.63 (0.42 to 0.97)	0.033
Re-hospitalization due to schizophrenia	9/123 (7.3%)	25/122 (20.5%)	0.36 (0.17 to 0.73)	0.004
Death for any reason	2/139 (1.4%)	1/134 (0.8%)	1.93 (0.18 to 21.01)	0.590
Substance abuse	14/133 (10.5%)	13/127 (10.2%)	1.028 (0.50 to 2.10)	0.939
Suicide	0/139 (0%)	0/139 (0%)	—	—
Self-harm	0/139 (0%)	0/139 (0%)	—	—
Wandering	2/138 (1.5%)	2/134 (1.5%)	0.97 (0.14 to 6.79)	0.976
Violence against others	1/137 (0.7%)	2/134 (1.5%)	0.49 (0.04 to 5.33)	0.557
Damaging goods	2/138 (1.5%)	5/134 (3.7%)	0.39 (0.08 to 1.97)	0.252

^a^Proportion of antipsychotic dosages taken over the past month assessed by unannounced home-based pill counts (possible range 0–1).

^b^Adjusted mean difference.

^c^Drug Attitude Inventory–10 (DAI-10) adherence was originally from −10 to +10 (higher score = more positive attitude toward medication), which was rescaled to be 0 to 1.

^d^Brief Adherence Rating Scale (BARS) is self-reported proportion of dosages taken over the past month.

^e^Refill record adherence is number of days medication obtained over past 182 days divided by 182 days.

^f^WHO Disability Assessment Schedule 2.0 (WHODAS): proportion of functioning lost (possible range 0–1).

^g^Clinical Global Impression (CGI)–Severity: Higher scores indicate worse symptoms (possible range 1–7).

^h^CGI–Improvement indicates degree of change in symptoms (1 = very much improved; 2 = much improved; 3 = minimally improved; 4 = no change; 5 = minimally worse; 6 = much worse; 7 = very much worse).

^i^These outcomes were tracked by the 686 Program administrative system on a routine basis. There was a small number of missing data.

^j^Relapse is defined as an overall and marked increase in symptoms as reassessed by health professionals through interviewing patients and family members.

### Secondary outcomes: Functioning and symptoms

There was slightly less loss of functioning in the intervention group than in the control group, though the difference was not statistically significant (mean WHODAS score 0.12 in the intervention group and 0.15 in the control group; AMD −0.03 [95% CI −0.07 to 0.01]; *p =* 0.117; effect size 0.18; Fig 2). There is, however, evidence of effect modification with baseline medication adherence (*p* for interaction 0.08): For the subset with good medication adherence at baseline, the mean WHODAS score was 0.19 in the intervention group and 0.09 in the control group (AMD −0.08 [95% CI −0.15 to −0.01]; *p =* 0.017; effect size 0.57; Fig 2); however, there was no significant difference in patient functioning between the groups for the subset with poor baseline adherence (AMD 0.01 [95% CI −0.04 to 0.07]; *p =* 0.603; Fig 2). We did not note any significant improvement in the severity of symptoms for the overall group or the pre-specified subgroups (Fig 2).

### Other outcomes

There was strong evidence of a substantial reduction in the risk of relapse (26 [21.7%] of 120 interventional participants versus 40 [34.2%] of 117 controls; relative risk [RR] 0.63 [95% CI 0.42 to 0.97]); number needed to treat [NNT] 8.0 [95% CI 4.2–85.2]) and the risk of re-hospitalization (9 [7.3%] of 123 interventional participants versus 25 [20.5%] of 122 controls; RR 0.36 [95% CI 0.17 to 0.73]; NNT 7.6 [95% CI 4.6–21.3]) with the intervention (Table 3).

### Raw versus adjusted analyses

We performed a sensitivity analysis to compare the results of the program effects on adherence, functioning, and symptoms with a raw analysis versus an adjusted analysis with covariates and data imputation for the missing data. The results were not sensitive to the choice of the methods, with almost identical results for adherence and functioning and a minor difference for symptoms (see S4 Appendix).

## Discussion

In this study, we used a 2-arm randomized controlled trial to study the effect of mobile texting on medication adherence, functioning, and symptoms of community-dwelling people with schizophrenia in rural China. Our trial showed that the addition of texting to patients and their lay health supporters in a resource-poor community setting compared with a free-medicine program alone improved medication adherence (0.48 in the control group versus 0.61 in the intervention group; effect size 0.38) and substantially reduced relapses and re-hospitalizations, but our program did not lead to significant changes in patient functioning or symptoms. The program was also found to be generally well accepted by the patients and their families, was relatively easy to implement and use, and added little marginal cost.

The existing evidence of the effect of texting on adherence, functioning, and symptoms is conflicting [15]. Six randomized controlled trials were identified that used texting for people with schizophrenia [15,50]: A recent trial in Finland (*n =* 1,139) showed no advantages of texting on any outcomes assessed at 12 months [51], which conformed to the results of 2 earlier trials (Netherlands [*n =* 62] [52]; Czech Republic [*n =* 146] [53]). One trial carried out in Spain (*n =* 254) [54] and 2 US trials (*n =* 30 and *n =* 55) [55,56], however, found significant improvement in medication adherence and some reduction in symptoms. Few studies reported adequately on outcomes related to patient functioning.

We would like to discuss 4 aspects of LEAN relative to prior studies. First, LEAN demonstrated a 27% relative improvement in adherence, which is larger than the 15%–18% range reported in other text message interventions [27]. Meanwhile, our program improved patients’ attendance at scheduled clinical appointments. Prior studies found mixed effects of the use of technological prompts on appointment attendance in psychological settings [57,58]. Three unique features may have contributed to the relative superiority of LEAN: (1) active engagement of the lay health supporters, (2) the varying contents of our medication reminder, which probably reduced receivers’ fatigue compared to other studies [59], and (3) the use of texting to connect and integrate the entire treatment team, from patients to the lay health supporters to the village doctors to the psychiatrists, all in support of the patient. In line with the theory of the HBM [33], text reminders and lay health supporters may have provided “cues to action” to address forgetfulness and reluctance to take medicine [30], while the texted education may have improved the perceived net benefits of the medications. There was improved attitude toward medication as shown by DAI-10 score, although the action cues probably played a bigger role—45.8% of patients regarded text medication reminders as most useful, while only 17.0% considered educational messages most useful.

Second, like most of the 6 randomized controlled trials discussed earlier [15,50], the improvement in medication adherence did not lead to significant reported changes in symptoms. Perhaps there was a ceiling effect, as the program participants in those studies and ours on average had only mild symptoms at baseline (Table 1). It may also be possible that the low adherence, even after LEAN, prevented the medicine from releasing its full potency. Even so, the substantial reduction in relapses (RR 0.63) and re-hospitalizations (RR 0.36) may indicate that, despite lack of effect for the whole group, there may be some program effect on symptoms for certain subsets of patients.

Third, prior studies of texting for schizophrenia seldom reported the global functioning level of the patients. Our program had a small and statistically insignificant effect on reported patient functioning for the overall group (effect size 0.17; *p =* 0.117), but it had a medium and significant effect for the subset with good baseline adherence (effect size 0.57; *p =* 0.017). As this improvement in the subset was not accompanied by improvement in medication adherence, we suspect that the text messages may have served as a rudimentary psychosocial intervention that was beneficial to functioning. Earlier studies indicated even simple messages asking “how are you?” or saying “thank you” reduced social isolation and improved functioning [60]. However, we should note that this subgroup effect may simply result from pure chance.

Finally, our program appears to have achieved a good level of participant satisfaction, and our program attrition was only 4.3%, compared with an overall rate of 20.0% (95% CI 17% to 24%) [61] in interventional trials for schizophrenia.

The trial used a waitlist control design whereby the control group would receive the intervention as well once the program demonstrated benefits after the initial 6-month implementation. We suspended LEAN from August 2017 to March 2018 due to our program evaluation. We resumed LEAN in both the original intervention and the waitlist control groups from April to October 2018. We are now in the process of cleaning the data from this extended program phase and will report the results in subsequent publications (program updates are available from https://www.researchgate.net/project/LEAN-Trial-Lay-Workers-mHealth-for-Severe-Mental-Disorders).

A special issue concerning the use of clozapine is worth discussion. Over 30% of our program participants used clozapine. Because of the close monitoring needed with this medication, the use of clozapine itself may increase treatment adherence and reduce symptoms. However, as the use of clozapine between our intervention and control groups was balanced at baseline (35% versus 33%) (Table 1), the use of clozapine should not lead to bias in our assessment of the program effects. Further, LEAN might help improve the use of clozapine because of the enhanced education on side effects and the facilitated communication between the patient families and the health professionals through texting, for quicker medication adjustment in between routine psychiatrists’ visits every 2 months.

Many lessons learned from this trial can be potentially useful for other LMICs that face resource constraints. China’s 686 Program successfully implemented many WHO mhGAP recommendations for resource-poor settings. In particular, the 686 Program in Liuyang effectively removed the access barriers for antipsychotics by providing free medication routinely and conveniently. However, adherence to antipsychotics remained a serious challenge. Our texting intervention further improved the program by addressing the low adherence at marginal cost. Elements of LEAN may be adapted to other resource-poor settings with or without an existing community-based program. However, adaptation of LEAN should fully consider some implementation details including (1) keeping the program simple and integrated into routine care [62,63] (LEAN required minimal training and leveraged existing resources such as a commercial telemarketing platform and the 686 Program structures), (2) maintaining low cost (LEAN cost a total of US$7,926 for the 139 patient participants and 139 lay health supporters), (3) having a reliable system to track changes of phone numbers (participants frequently changed numbers [Table 3]), and (4) choosing the right phones (some cheap phones’ small storage filled up quickly and prevented incoming messages). Furthermore, long-acting injectable antipsychotics were not available through the 686 Program. Both the clinicians and families perceived the injectable to be unpredictable and less safe. The 686 Program should develop a guideline on the use of those long-acting injectables, particularly among people with low adherence to pills. We should also note that adherence measured by clinician impression and refill records (the current 686 Program practice) grossly overestimated the level of adherence; the use of simple scales such as DAI-10 or BARS may be considered if home-based pill counts are not feasible [44]. Lastly, we emphasize proper training for the participants on how to receive, read, and reply to text messages. Despite our training, at the endpoint, 28.8% of patients still could not read the messages. To address this challenge, we sent voice messages for some participants and simultaneously texted the lay health supporters. Our experiences caution against the use of more complicated smartphones among people with schizophrenia with low education in LMICs.

There are several limitations to our trial. First, certain features of the 686 Program may limit the generalizability of the findings to other parts of the world, given that other populations may have limited access to medications, or distinct cultural beliefs about the origins and meaning of mental illness. However, the study should provide solid reference points for programs considering the use of texting and lay health supporters. Second, we investigated our program’s cost, but we did not perform a cost-effectiveness analysis. This partially reflected the preference of the local policy-makers for low cost rather than cost-effectiveness. Third, our pursuit of simplicity sacrificed the ability to customize the content, frequency, and timing of the messages to individual patients. Individual tailoring is considered more effective [15,17] but would have significantly increased program complexity and cost. Fourth, our trial only had a 6-month follow-up, and thus we could not determine the longer-term effects of the intervention on adherence, symptoms, and functioning. Fifth, despite our best efforts to capture adherence, the unannounced pill count can still be subject to inaccuracy. In particular, the number of discarded and additionally obtained pills as reported by the patients and their family members may be inaccurate because of memory lapses or by intention. However, this possible inaccuracy may not lead to bias as its effects may be canceled out between the intervention and control groups due to randomization. Sixth, due to a program administrative mishap, we failed to assess medication side effects at endpoint as our protocol had specified. We thus had no information on the effect of LEAN on medication side effects. Seventh, we could assess the overall effects of LEAN on adherence, functioning, and symptoms, but the effects could not be attributed to specific program elements (e.g., how much of the program’s effects can be attributed to our text medication reminders to the lay health supporters or the patients?) Eighth, there was a possible risk of bias if family members were present during our patient assessment in a different fashion between the 2 groups. We tried hard to stick to the same assessment protocol to reduce this risk. Finally, our program possibly missed some of the least adherent people as we excluded those missing the 3 past medication refills. This de facto withdrawal from the 686 Program may be for reasons such as feeling highly functioning and deciding to discontinue medications, choosing to purchase medications outside of the 686 Program, or intentionally or unintentionally missing refills due to sickness.

Our study points to several future directions for research. Some non-schizophrenia studies have suggested that less frequent messages are more effective [15]. Future trials should test that possibility. Furthermore, 33.3% of lay health supporters and 29.0% of patients used a smartphone. Smartphones, with their sensor technologies and apps, may have considerable potential for improving the health of people with schizophrenia [64]. However, complicated apps may create barriers as well, considering that 28.8% and 47.9% of our patients could not even master the simple task of reading and replying to messages, respectively. The role of smartphones needs to be further explored in trials. Finally, potential adverse effects of text messaging on patients and their families should be more thoroughly investigated. Four (6.3%) patients and 10 (10.7%) lay health supporters did report texting bothered them.

## Supporting information

S1 CONSORT CONSORT checklist. (DOC)

S1 Appendix Multiple imputation used in our statistical analysis. (DOCX)

S2 Appendix Sample text messages. (DOCX)

S3 Appendix Free medications dispensed in the 686 Program in Liuyang, Hunan, China. (DOCX)

S4 Appendix Raw analysis versus adjusted analysis with covariates and data imputation. (DOCX)

## Abbreviations

AMD: adjusted mean difference
BARS: Brief Adherence Rating Scale
CGI: Clinical Global Impression
DAI-10: Drug Attitude Inventory–10
GEE: generalized estimating equation
HBM: health belief model
LMICs: low- and middle-income countries
mHealth: mobile health
mhGAP: Mental Health Gap Action Programme
NNT: number needed to treat
RR: relative risk
THC: township health center
WHODAS: WHO Disability Assessment Schedule 2.0

## References

[1] Saha S, Chant D, Welham J, McGrath J. A systematic review of the prevalence of schizophrenia. PLoS Med. 2005;2(5):e141 10.1371/journal.pmed.0020141 15916472 PMC1140952

[2] Institute for Health Metrics and Evaluation. GBD compare Seattle: Institute for Health Metrics and Evaluation; 2019 [cited 2019 Mar 26]. Available from: http://www.healthdata.org/data-visualization/gbd-compare.

[3] Rice DP, Miller LS. The economic burden of schizophrenia: conceptual and methodological issues, and cost estimates In: Moscarelli M, Rupp A, Sartorius N, editors. Handbook of mental health economics and health policy. Vol. 1. Schizophrenia. Oxford: John Wiley & Sons; 1996 pp. 321–34.

[4] Chan M. Mental health and development: targeting people with mental health conditions as a vulnerable group Geneva: World Health Organization; 2010 [cited 2019 Jan 28]. Available from: https://www.who.int/mental_health/policy/mhtargeting/en/.

[5] World Health Organization. mhGAP: Mental Health Gap Action Programme—scaling up care for mental, neurological and substance use disorders Geneva: World Health Organization; 2008 [cited 2019 Jan 28]. Available from: https://www.who.int/mental_health/mhgap_final_english.pdf?ua=1. 26290926

[6] Kohn R, Saxena S, Levav I, Saraceno B. The treatment gap in mental health care. Bull World Health Organ. 2004;82(11):858–66. 15640922 PMC2623050

[7] Patel V, Xiao S, Chen H, Hanna F, Jotheeswaran A, Luo D, et al The magnitude of and health system responses to the mental health treatment gap in adults in India and China. Lancet. 2016;388(10063):3074–84. 10.1016/S0140-6736(16)00160-4 27209149

[8] McBain R, Norton DJ, Morris J, Yasamy MT, Betancourt TS. The role of health systems factors in facilitating access to psychotropic medicines: a cross-sectional analysis of the WHO-AIMS in 63 low-and middle-income countries. PLoS Med. 2012;9(1):e1001166 10.1371/journal.pmed.1001166 22303288 PMC3269418

[9] Barkhof E, Meijer CJ, de Sonneville LM, Linszen DH, de Haan L. Interventions to improve adherence to antipsychotic medication in patients with schizophrenia—a review of the past decade. Eur Psychiatry. 2012;27(1):9–18. 10.1016/j.eurpsy.2011.02.005 21561742

[10] DiMatteo MR. Variations in patients’ adherence to medical recommendations: a quantitative review of 50 years of research. Med Care. 2004;42(3):200–9. 15076819 10.1097/01.mlr.0000114908.90348.f9

[11] Saxena S, Thornicroft G, Knapp M, Whiteford H. Resources for mental health: scarcity, inequity, and inefficiency. Lancet. 2007;370(9590):878–89. 10.1016/S0140-6736(07)61239-2 17804062

[12] Collins PY, Patel V, Joestl SS, March D, Insel TR, Daar AS, et al Grand challenges in global mental health. Nature. 2011;475(7354):27–30. 10.1038/475027a 21734685 PMC3173804

[13] Shidhaye R, Lund C, Chisholm D. Closing the treatment gap for mental, neurological and substance use disorders by strengthening existing health care platforms: strategies for delivery and integration of evidence-based interventions. Int J Ment Health Syst. 2015;9(1):40. 26719762 10.1186/s13033-015-0031-9 PMC4696279

[14] Fiordelli M, Diviani N, Schulz PJ. Mapping mHealth research: a decade of evolution. J Med Internet Res. 2013;15(5):e95 10.2196/jmir.2430 23697600 PMC3668610

[15] Berrouiguet S, Baca-García E, Brandt S, Walter M, Courtet P. Fundamentals for future mobile-health (mHealth): a systematic review of mobile phone and web-based text messaging in mental health. J Med Internet Res. 2016;18(6):e135 10.2196/jmir.5066 27287668 PMC4920962

[16] Braun R, Catalani C, Wimbush J, Israelski D. Community health workers and mobile technology: a systematic review of the literature. PLoS ONE. 2013;8(6):e65772 10.1371/journal.pone.0065772 23776544 PMC3680423

[17] Hall AK, Cole-Lewis H, Bernhardt JM. Mobile text messaging for health: a systematic review of reviews. Annu Rev Public Health. 2015;36:393–415. 10.1146/annurev-publhealth-031914-122855 25785892 PMC4406229

[18] Head KJ, Noar SM, Iannarino NT, Harrington NG. Efficacy of text messaging-based interventions for health promotion: a meta-analysis. Soc Sci Med. 2013;97:41–8. 10.1016/j.socscimed.2013.08.003 24161087

[19] Neville R, Charnock Greene A, McLeod J, Tracey A, Surie J. Mobile phone text messaging can help young people with asthma. BMJ. 2002;325(7364):600 10.1136/bmj.39552.645775 PMC1124119 12228151

[20] Gurol-Urganci I, de Jongh T, Vodopivec-Jamsek V, Atun R, Car J. Mobile phone messaging reminders for attendance at healthcare appointments. Cochrane Database Syst Rev. 2013;12:CD007458. 10.1002/14651858.CD007458.pub3 PMC6485985 24310741

[21] Atherton H, Sawmynaden P, Meyer B, Car J. Email for the coordination of healthcare appointments and attendance reminders. Cochrane Database Syst Rev. 2012;8:CD007981 10.1002/14651858.CD007981.pub2 22895971 PMC11627138

[22] Guy R, Hocking J, Wand H, Stott S, Ali H, Kaldor J. How effective are short message service reminders at increasing clinic attendance? A meta‐analysis and systematic review. Health Serv Res. 2012;47(2):614–32. 10.1111/j.1475-6773.2011.01342.x 22091980 PMC3419880

[23] Kauppi K, Välimäki M, Hätönen HM, Kuosmanen LM, Warwick‐Smith K, Adams CE. Information and communication technology based prompting for treatment compliance for people with serious mental illness. Cochrane Database Syst Rev. 2014;6:CD009960 10.1002/14651858.CD009960.pub2 24934254 PMC8078321

[24] Bright CE. Integrative review of mobile phone contacts and medication adherence in severe mental illness. J Am Psychiatr Nurses Assoc. 2018;24(3):209–22. 10.1177/1078390318754986 29457508

[25] Naslund JA, Marsch LA, McHugo GJ, Bartels SJ. Emerging mHealth and eHealth interventions for serious mental illness: a review of the literature. J Ment Health. 2015;24(5):321–32. 10.3109/09638237.2015.1019054 26017625 PMC4924808

[26] Firth J, Cotter J, Torous J, Bucci S, Firth JA, Yung AR. Mobile phone ownership and endorsement of “mHealth” among people with psychosis: a meta-analysis of cross-sectional studies. Schizophr Bull. 2015;42(2):448–55. 10.1093/schbul/sbv132 26400871 PMC4753601

[27] DeKoekkoek T, Given B, Given CW, Ridenour K, Schueller M, Spoelstra SL. mHealth SMS text messaging interventions and to promote medication adherence: an integrative review. J Clin Nurs. 2015;24(19–20):2722–35. 10.1111/jocn.12918 26216256

[28] Tian M, Zhang J, Luo R, Chen S, Petrovic D, Redfern J, et al mHealth interventions for health system strengthening in China: a systematic review. JMIR Mhealth Uhealth. 2017;5(3):e32 10.2196/mhealth.6889 28302597 PMC5374274

[29] Riley WT, Rivera DE, Atienza AA, Nilsen W, Allison SM, Mermelstein R. Health behavior models in the age of mobile interventions: are our theories up to the task? Transl Behav Med. 2011;1(1):53–71. 10.1007/s13142-011-0021-7 21796270 PMC3142960

[30] Xu DR, Gong W, Caine ED, Xiao S, Hughes JP, Ng M, et al Lay health supporters aided by a mobile phone messaging system to improve care of villagers with schizophrenia in Liuyang, China: protocol for a randomised control trial. BMJ Open. 2016;6(1):e010120 10.1136/bmjopen-2015-010120 26792221 PMC4735204

[31] World Health Organization. The ICD-10 classification of mental and behavioural disorders: clinical descriptions and diagnostic guidelines. Geneva: World Health Organization; 1992.

[32] Gong W, Xu D, Zhou L, Brown HS 3rd, Smith KL, Xiao S. Village doctor-assisted case management of rural patients with schizophrenia: protocol for a cluster randomized control trial. Implement Sci. 2014;9(1):13. 24433461 10.1186/1748-5908-9-13 PMC3929227

[33] Becker MH. The health belief model and personal health behavior. Health Educ Monogr. 1974;2:324–473.

[34] Lamberti JS. Seven keys to relapse prevention in schizophrenia. J Psychiatr Pract. 2001;7(4):253–9. 15990532 10.1097/00131746-200107000-00006

[35] Zhao M, Gong W, Xu D, Huang ZP, Hu M, Nie J, et al [The training effect on text-messaging skills of people with schizophrenia in rural China and its influencing factors.] China J Health Psychol. 2018;10:3.

[36] XinjuaNet News. [Health and Family Planning Commission: 5.4 million people with psychosis.] XinhuaNet. 2017 Apr 7 [cited 2017 Oct 7]. Available from: http://news.xinhuanet.com/politics/2017-04/07/c_129527228.htm.

[37] Nieuwlaat R, Wilczynski N, Navarro T, Hobson N, Jeffery R, Keepanasseril A, et al Interventions for enhancing medication adherence. Cochrane Database Syst Rev. 2014;11:CD000011 10.1002/14651858.CD000011.pub4 25412402 PMC7263418

[38] Bellack AS, Bowden CL, Bowie CR, Byerly MJ, Carpenter WT, Copeland LA, et al The expert consensus guideline series: adherence problems in patients with serious and persistent mental illness. J Clin Psychiatry. 2009;70(Suppl 4):1–48. 19686636

[39] Velligan DI, Lam Y-WF, Glahn DC, Barrett JA, Maples NJ, Ereshefsky L, et al Defining and assessing adherence to oral antipsychotics: a review of the literature. Schizophr Bull. 2006;32(4):724–42. 10.1093/schbul/sbj075 16707778 PMC2632258

[40] Velligan DI, Weiden PJ, Sajatovic M, Scott J, Carpenter D, Ross R, et al Assessment of adherence problems in patients with serious and persistent mental illness: recommendations from the Expert Consensus Guidelines. J Psychiatr Pract. 2010;16(1):34–45. 10.1097/01.pra.0000367776.96012.ca 20098229

[41] McCambridge J, Witton J, Elbourne DR. Systematic review of the Hawthorne effect: new concepts are needed to study research participation effects. J Clin Epidemiol. 2014;67(3):267–77. 10.1016/j.jclinepi.2013.08.015 24275499 PMC3969247

[42] Byerly MJ, Nakonezny PA, Rush AJ. The Brief Adherence Rating Scale (BARS) validated against electronic monitoring in assessing the antipsychotic medication adherence of outpatients with schizophrenia and schizoaffective disorder. Schizophr Res. 2008;100(1):60–9. 18255269 10.1016/j.schres.2007.12.470

[43] Hogan TP, Awad A, Eastwood R. A self-report scale predictive of drug compliance in schizophrenics: reliability and discriminative validity. Psychol Med. 1983;13(1):177–83. 6133297 10.1017/s0033291700050182

[44] Xu DR, Gong W, Gloyd S, Caine ED, Simoni J, Hughes JP, et al Measuring adherence to antipsychotic medications for schizophrenia: concordance and validity among a community sample in rural China. Schizophr Res. 2018;201:307–14. 10.1016/j.schres.2018.05.014 29807806 PMC6252110

[45] Haro J, Kamath S, Ochoa S, Novick D, Rele K, Fargas A, et al The Clinical Global Impression–Schizophrenia scale: a simple instrument to measure the diversity of symptoms present in schizophrenia. Acta Neurol Scand. 2003;107(s416):16–23. 10.1034/j.1600-0447.107.s416.5.x 12755850

[46] Üstün TB, Kostanjesek N, Chatterji S, Rehm J, editors. Measuring health and disability: manual for WHO Disability Assessment Schedule (WHODAS 2.0) Geneva: World Health Organization; 2010.

[47] Harris PA, Taylor R, Thielke R, Payne J, Gonzalez N, Conde JG. Research electronic data capture (REDCap)—a metadata-driven methodology and workflow process for providing translational research informatics support. J Biomed Inform. 2009;42(2):377–81. 10.1016/j.jbi.2008.08.010 18929686 PMC2700030

[48] van Buuren S. Package ‘mice’. Vienna: Comprehensive R Archive Network; 2015.

[49] Cohen J. Statistical power analysis for the behavioral sciences. 2nd edition Hilsdale (NJ): Lawrence Earlbaum Associates; 1988.

[50] El-Mallakh P, Findlay J. Strategies to improve medication adherence in patients with schizophrenia: the role of support services. Neuropsychiatr Dis Treat. 2015;11:1077 10.2147/NDT.S56107 25931823 PMC4404876

[51] Välimäki M, Kannisto KA, Vahlberg T, Hätönen H, Adams CE. Short text messages to encourage adherence to medication and follow-up for people with psychosis (Mobile. Net): randomized controlled trial in Finland. J Med Internet Res. 2017;19(7):e245 10.2196/jmir.7028 28701292 PMC5529737

[52] Pijnenborg G, Withaar F, Brouwer WH, Timmerman M, Bosch R, Evans J. The efficacy of SMS text messages to compensate for the effects of cognitive impairments in schizophrenia. Br J Health Psychol. 2010;49(2):259–74. 10.1348/014466509X467828 19735607

[53] Španiel F, Hrdlicka J, Novák T, Kožený J, Hoeschl C, Mohr P, et al Effectiveness of the information technology-aided program of relapse prevention in schizophrenia (ITAREPS): a randomized, controlled, double-blind study. J Psychiatr Pract. 2012;18(4):269–80. 10.1097/01.pra.0000416017.45591.c1 22805901

[54] Montes JM, Medina E, Gomez-Beneyto M, Maurino J. A short message service (SMS)-based strategy for enhancing adherence to antipsychotic medication in schizophrenia. Psychiatry Res. 2012;200(2):89–95. 22901437 10.1016/j.psychres.2012.07.034

[55] Beebe L, Smith KD, Phillips C. A comparison of telephone and texting interventions for persons with schizophrenia spectrum disorders. Issues Ment Health Nurs. 2014;35(5):323–9. 10.3109/01612840.2013.863412 24766166

[56] Granholm E, Ben-Zeev D, Link PC, Bradshaw KR, Holden JL. Mobile Assessment and Treatment for Schizophrenia (MATS): a pilot trial of an interactive text-messaging intervention for medication adherence, socialization, and auditory hallucinations. Schizophr Bull. 2012;38(3):414–25. 10.1093/schbul/sbr155 22080492 PMC3329971

[57] Clough BA, Casey LM. Using SMS reminders in psychology clinics: a cautionary tale. Behav Cogn Psychother. 2014;42(03):257–68. 24495365 10.1017/S1352465813001173

[58] Thomas IF, Lawani AO, James BO. Effect of short message service reminders on clinic attendance among outpatients with psychosis at a psychiatric hospital in Nigeria. Psychiatr Serv. 2016;68(1):75–80. 10.1176/appi.ps.201500514 27582239

[59] Strandbygaard U, Thomsen SF, Backer V. A daily SMS reminder increases adherence to asthma treatment: a three-month follow-up study. Respir Med. 2010;104(2):166–71. 10.1016/j.rmed.2009.10.003 19854632

[60] Agyapong VI, McLoughlin DM, Farren CK. Six-months outcomes of a randomised trial of supportive text messaging for depression and comorbid alcohol use disorder. J Affect Disord. 2013;151(1):100–4. 10.1016/j.jad.2013.05.058 23800443

[61] Szymczynska P, Walsh S, Greenberg L, Priebe S. Attrition in trials evaluating complex interventions for schizophrenia: systematic review and meta-analysis. J Psychiatr Res. 2017;90:67–77. 10.1016/j.jpsychires.2017.02.009 28231496

[62] Priebe S, Kelley L, Omer S, Golden E, Walsh S, Khanom H, et al The effectiveness of a patient-centred assessment with a solution-focused approach (DIALOG+) for patients with psychosis: a pragmatic cluster-randomised controlled trial in community care. Psychother Psychosom. 2015;84(5):304–13. 10.1159/000430991 26278784

[63] O’Hanlon P, Aref-Adib G, Fonseca A, Lloyd-Evans B, Osborn D, Johnson S. Tomorrow’s world: current developments in the therapeutic use of technology for psychosis. BJPsych Adv. 2016;22(5):301–10.

[64] Firth J, Torous J. Smartphone apps for schizophrenia: a systematic review. JMIR Mhealth Uhealth. 2015;3(4):e102 10.2196/mhealth.4930 26546039 PMC4704940

