# Supplementary material for: Lay health supporters aided by mobile text messaging to improve adherence, symptoms, and functioning among people with schizophrenia in a resource-poor community in rural China (LEAN): A randomized controlled trial
Source: PLoS Med. 2019 Apr 23;16(4):e1002785. doi: 10.1371/journal.pmed.1002785 (PMC6478272; doi:10.1371/journal.pmed.1002785)
Supplement: S1 Appendix — (DOCX) [file pmed.1002785.s002.docx]

S1 Appendix: Multiple imputation used in our statistical analysis

In the analysis process, we mainly use the R package mice, gee, norm. (MICE: Multivariate Imputation by Chained Equations). In the multiple imputations, we imputed 10 complete datasets. The GEE model is then applied for each dataset, and the synthetization of 10 GEE model results is done with the norm R package. We describe the specific steps below, using the analysis of the effect of the program on medication adherence:

1. Assessing the missing data pattern: We visualized the pattern of missing data in order to discern any systematic pattern of missing (Fig 1 and Fig 2). As we did not find any clear pattern, we assume that data are missing at random in our analysis.
2. Selecting the data imputation model: If the variable with missing value is a continuous variable, the predictive mean matching (PMM) method is used; if the variable with missing value is a binary variable, Logistic regression is then used; while for a multi-level categorical missing variable, the polytomous regression is applied..
3. Determining the independent variables used in the imputation model: all other variables except for the dependent variable were used in the imputation model. In order to improve the accuracy of the imputation, in addition to the variables used for the GEE model including the variables of intervention assignment, adherence, functioning, substance use, drug side effects, family supervision, we also used other social-economic and demographic variables such as sex, age, education, income, living alone, duration of illness, symptoms severity etc.in the imputation model.
4. Determining the order of imputation: From left to right: demographic/social-economic information and other variables, the independent variables used in the GEE model, dependent variables used in the GEE model.
5. Ten complete datasets generated were generated: Fig 3 shows the original observed data and the imputed data. The imputed data sets show a similar pattern of data distribution to the original dataset.
6. GEE model was performed for each of the 10 complete data sets.
7. Using norm R package to synthetize the results from the 10 GEE models to obtain the overall estimate of the effect of the program.

*References: MICE: Multivariate Imputation by Chained Equations in R*


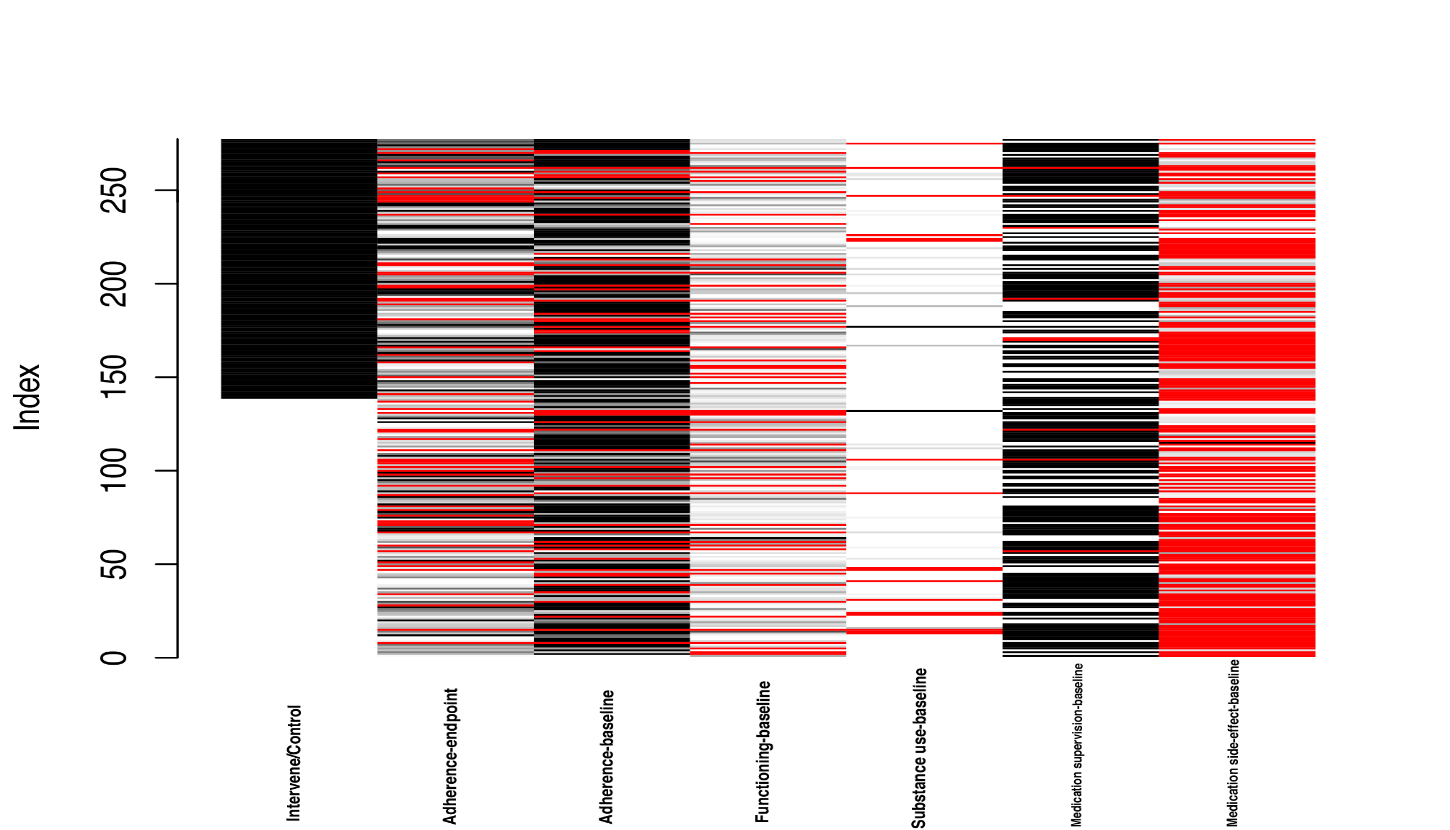
Fig 1: missing data comparison: control vs. intervention groups; Red indicting missing; gray and black indicating available data; darker color indicting greater value.


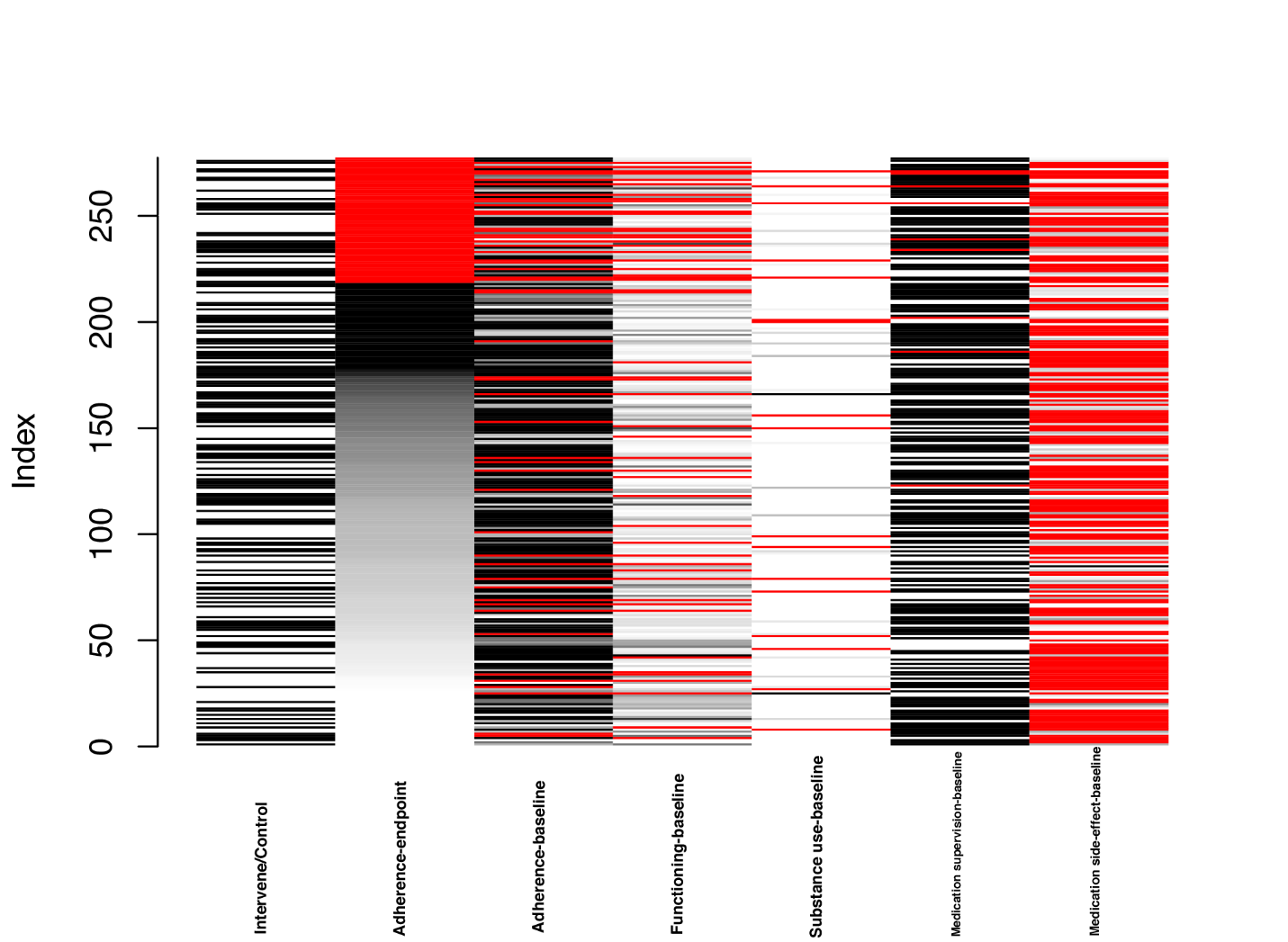


Fig 2: Missing data (sorted in order of medication adherence level)


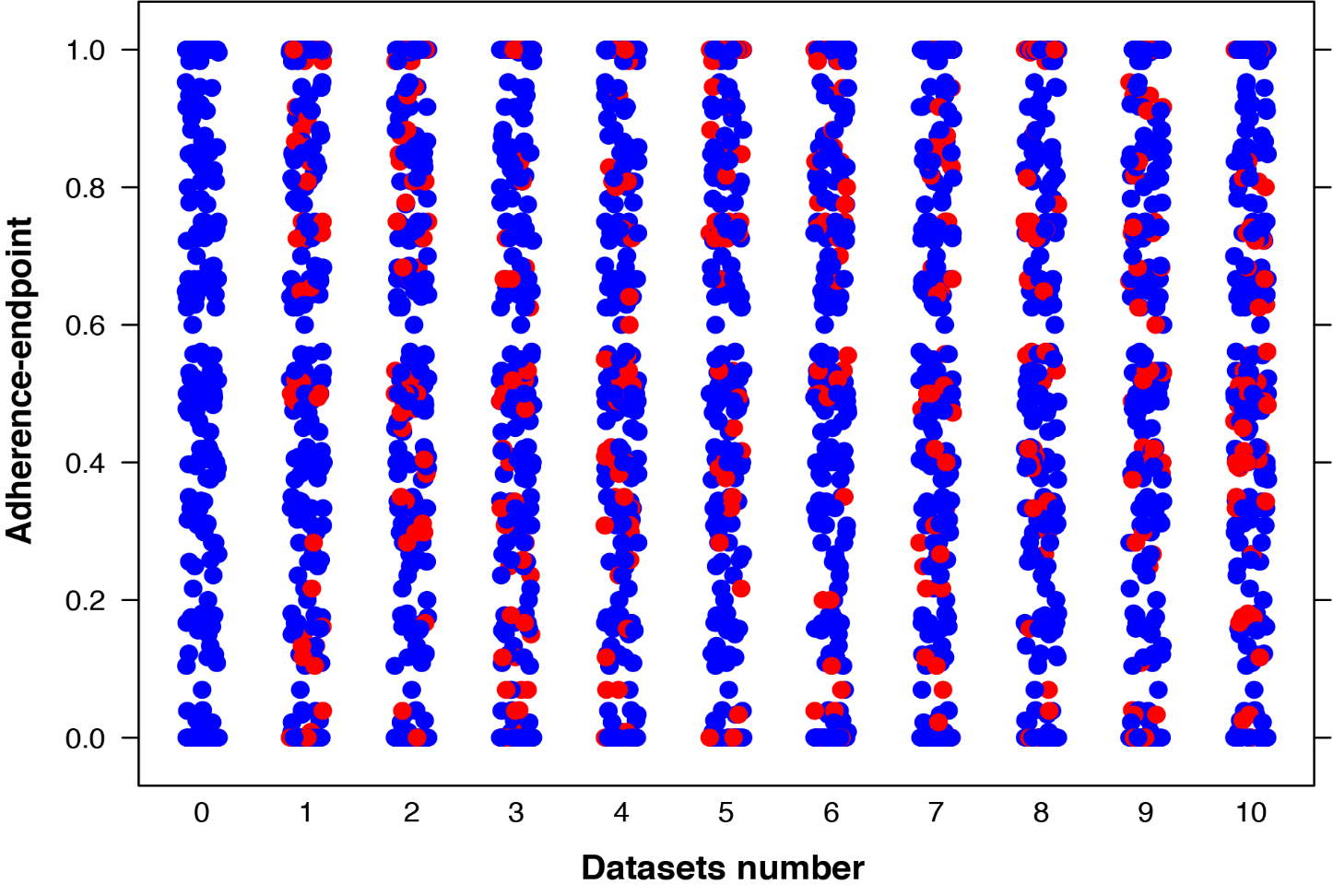


Fig 3 Imputed datasets, using medication adherence as an example; 0 represents the original observed data; 1-10 represent datasets after multiple imputation, with the blue dots indicating the original observed values, the red dots representing the imputed values
