# Supplementary material for: Lay health supporters aided by mobile text messaging to improve adherence, symptoms, and functioning among people with schizophrenia in a resource-poor community in rural China (LEAN): A randomized controlled trial
Source: PLoS Med. 2019 Apr 23;16(4):e1002785. doi: 10.1371/journal.pmed.1002785 (PMC6478272; doi:10.1371/journal.pmed.1002785)
Supplement: S2 Appendix — (DOCX) [file pmed.1002785.s003.docx]

S2 Appendix: Sample text messages

1. **Daily medication reminder:** Good evening! Temple fair in Baisheng town tomorrow and good weather. Please take your medicine and text back “1”.
2. **Education message** (to the lay health supporters): Note any lack of interests in things that they used to like as it may be early sign of relapse. We are with you, caring for the patients.
3. **Education message** (to patients): Adhering to your medication on time and on the prescribed dose is the key to control your symptoms. We are here to help you.
4. **Education message** (to the lay health supporters): People with schizophrenia may hear voices not heard by others, or think others can see their thoughts, control their own thinking, or attempt to harm themselves. Those can lead to fear, withdrawal or emotional agitation. Try to understand them and get their trust.
5. **Monitoring messages**: Text 1 if any of the following happens or worsens: problem with sleep, appetite, or concentration; depression; restlessness; tension or nervousness; hearing voices or seeing things that others can’t hear or see; less pleasure gained from things you enjoy; feeling people were watching you; preferring being alone; arguments with others; inability to get your mind off of something.
