# Supplementary material for: Lay health supporters aided by mobile text messaging to improve adherence, symptoms, and functioning among people with schizophrenia in a resource-poor community in rural China (LEAN): A randomized controlled trial
Source: PLoS Med. 2019 Apr 23;16(4):e1002785. doi: 10.1371/journal.pmed.1002785 (PMC6478272; doi:10.1371/journal.pmed.1002785)
Supplement: S3 Appendix — (DOCX) [file pmed.1002785.s004.docx]

S3 Appendix: Free medications dispensed at the 686 Program in Liuyang, Hunan, China

| Drug | All (n=269) | Intervene (n=136) | Control (n=133) | *P value* |
| --- | --- | --- | --- | --- |
|  | n/N(%) | n/N(%) | n/N(%) |  |
| [**clozapine**](C:/Users/Administrator/AppData/Local/youdao/dict/Application/6.3.69.8341/resultui/frame/javascript:void(0);) | 93/269(34.6%) | 48/136(35.3%) | 45/133(33.8%) | 0.801 |
| **risperidone** | 89/269(33.1%) | 46/136(33.8%) | 43/133(32.3%) | 0.795 |
| **quetiapine** | 51/269(19.0%) | 26/136(19.1%) | 25/133(18.8%) | 0.947 |
| **sulpiride** | 45/269(16.7%) | 21/136(15.4%) | 25/133(18.8%) | 0.176 |
| **perphenazine** | 27/269(10.0%) | 12/136(8.8%) | 15/133(11.3%) | 0.017 |
| **aripiprazole** | 17/269(6.3%) | 5/136(3.7%) | 12/133(9.0%) | 0.072 |
| **chlorpromazine** | 13/269(4.8%) | 10/136(7.4%) | 3/133(2.3%) | 0.051 |
| **olanzapine** | 12/269(4.5%) | 5/136(3.7%) | 7/133(5.3%) | 0.529 |
| **perphenazine[injection]** | 9/269(3.3%) | 6/136(4.4%) | 3/133(2.3%) | 0.520 |
| **penfluridol** | 5/269(1.9%) | 3/136(2.2%) | 2/133(1.5%) | 1.000 |
| **haloperidol** | 3/269(1.1%) | 2/136(1.5%) | 1/133(0.8%) | 1.000 |
| **trifluoperazine** | 1/269(0.4%) | 1/136(0.7%) | 0/133(0.0%) | 1.000 |
