# Supplementary material for: Lay health supporters aided by mobile text messaging to improve adherence, symptoms, and functioning among people with schizophrenia in a resource-poor community in rural China (LEAN): A randomized controlled trial
Source: PLoS Med. 2019 Apr 23;16(4):e1002785. doi: 10.1371/journal.pmed.1002785 (PMC6478272; doi:10.1371/journal.pmed.1002785)
Supplement: S4 Appendix — (DOCX) [file pmed.1002785.s005.docx]

**S4 Appendix**: Raw versus Adjusted Analysis with covariates and Data Imputation

|  |  | **Raw Analysis** | |  | **Adjusted Analysis**  **with covariates and data imputation** | |
| --- | --- | --- | --- | --- | --- | --- |
| **Measures** |  | **Mean difference(95%CI) or relative risk(95%CI)** | **P value** |  | **Mean difference(95%CI) or relative risk(95%CI)** | **P value** |
| Pill-count adherence |  | 0.13(0.04 to 0.22) | 0.007 |  | 0.12(0.03 to 0.22) | 0.013 |
| WHODAS |  | -0.03(-0.07 to 0.02) | 0.204 |  | -0.03(-0.07 to 0.01) | 0.117 |
| CGI-severity of illness |  | 0.09(-0.24 to 0.41) | 0.595 |  | 0.11(-0.21 to 0.42) | 0.514 |
| CGI-degree of change |  | 0.07(-0.21 to 0.34) | 0.644 |  | 0.03(-0.25 to 0.30) | 0.848 |
